# Optimization of ultrasonic assisted ethanolic extraction for natural pigments from butterfly pea flower applied in Thai dessert using Box-Behnken approach

**DOI:** 10.1016/j.fochx.2024.101484

**Published:** 2024-05-17

**Authors:** Samart Sai-Ut, Apisara Teksee, Jaksuma Pongsetkul, Sirima Sinthusamran, Saroat Rawdkuen

**Affiliations:** a Department of Food Science, Faculty of Science, Burapha University, Chonburi, Thailand; b School of Animal Technology and Innovation, Institute of Agricultural Technology, Suranaree University of Technology, Nakhon Ratchasima, Thailand; c Department of Agricultural Education, School of Industrial Education and Technology, King Mongkut's Institute of Technology Ladkrabang, Bangkok, Thailand; d Food Science and Technology Program, School of Agro-Industry, Mae Fah Luang University, Chiang Rai, Thailand

**Keywords:** Butterfly pea flower, Colorant, Response surface methodology, Ultrasound

## Abstract

Butterfly pea is a natural color source used in food and dessert. This study optimized ultrasound-assisted extraction with ethanol for pigments from butterfly pea flowers (BPF) using a Box-Behnken method. Key factors explored were solid-to-solvent ratio, ultrasound extraction time, and ethanol concentration. The extracted compounds were evaluated for extraction yield (EY), total phenolic content (TPC), total anthocyanin content (TAC), and DPPH antioxidant activity. EY increased significantly with reduced ethanol concentration. Optimal conditions were predicted and experimentally validated. BPF extracts showed distinct absorption wavelengths at different pH levels. BPF extract was used in coconut milk jelly, resulting in the lowest b* value. These findings highlight the value of optimal ultrasonic-assisted extraction for enhancing BPF's natural colorant extraction and promoting sustainable use in food and dessert applications.

## Highlights

• Ultrasonic extraction efficiently extracts polyphenols and antioxidants from BPF using cavitation.
• BPF extracts show color changes based on pH, affected by storage duration.
• Box-Behnken design optimizes BPF colorant extraction, enhancing vibrancy for Thai desserts.

## 1 Introduction

Color plays a significant role in the acceptability and attractiveness of food, often influencing consumers' perceptions of taste and quality. Consumer acceptance and perception of food quality are linked to color attributes. The rising consumer demand for natural ingredients and concerns related to nutrition and health have driven the adoption of natural color sources in the food and beverage sector. Natural colors in plants come from various pigments: red and yellow hues come from betalains, green from chlorophyll, red and purple from anthocyanins, and yellow and orange from carotenoids (Ghosh, Sarkar, Das, & Chakraborty, 2022). Natural pigments originating from edible fruits, vegetables, and flowers are not only perceived as safe and clean but are also acknowledged for their potential health benefits when utilized as food ingredients (Ghosh, Sarkar, Das, & Chakraborty, 2021).

Butterfly pea flowers (BPF), known as botanic name *Clitoria ternatea*, part of the Fabaceae family, are a natural source of food coloring. BPF serve culinary and medicinal purposes. Their extract is used as a natural food colorant, and their roots and leaves are used in herbal drinks. Butterfly pea powder, derived from the blue pea flower, is a well-known product. BPF contains various bioactive compounds, including anthocyanins, flavonoids, glycosides, steroids, resins, and phenols. These compounds contribute to its anti-diabetic, antioxidant, antibacterial, anti-inflammatory, and analgesic properties (Chusak et al., 2019). Acylated anthocyanins in *C. ternatea* are known for their structural complexity and stability, which makes them attractive for use as natural colorants in the food and beverage industry (Giusti & Wrolstad, 2003; Jeyaraj, Lim, & Choo, 2022). The acylation provides structural stability and impacts their color properties, making them more resistant to degradation by light, heat, and pH changes compared to non-acylated anthocyanins (Jokioja, Yang, & Linderborg, 2021). The traditional Thai dessert known as butterfly pea coconut milk jelly, renowned for its historical significance and widespread popularity, incorporates vibrant colors derived from butterfly pea flowers. The increasing demand for utilizing this natural extract in industrial-scale production underscores the necessity for an extraction process that is not only suitable but also aligns with the requirements of both producers and consumers.

Extraction of plant pigments using solvents is well-reported and simple, but it has disadvantages such as long exposure times and lower yields. Consequently, there is significant interest in “green” extraction technologies, which offer shorter processing times, higher yields, and lower environmental risks, addressing the limitations of conventional methods like Soxhlet and heat-assisted extraction. Ultrasonic assisted extraction (UAE) is a promising technology for efficiently retrieving natural pigments from by-products (Linares & Rojas, 2022). This approach involves the utilization of sound waves with specific properties, including frequency, amplitude, and wavelength, to induce physicochemical alterations in the medium. Ultrasonic transducers produce these waves by converting electrical energy into sound waves characterized by their desired frequency and intensity (Kentish, 2017). UAE offers multiple advantages, such as reduced extraction and processing duration, lower energy consumption, decreased carbon dioxide emissions, and minimized solvent usage. These benefits result in enhanced extraction yields and the potential for employing cleaner and environmentally friendly extraction processes, particularly with water or solvents (Chemat et al., 2017). Moreover, UAE optimizes the extraction of heat-sensitive components that might otherwise yield unsatisfactory results. However, UAE also has limitations. It can require significant energy, especially at higher power, leading to higher costs. Prolonged use generates heat, potentially degrading heat-sensitive compounds like anthocyanins, thus necessitating temperature control. The initial investment in ultrasonic equipment is high, and its operation requires specialized knowledge (Kumar, Srivastav, & Sharanagat, 2020). Scaling up to industrial scale is challenging due to variations in ultrasound transmission and cavitation in larger volumes. Intense ultrasonic power can break down bioactive compounds, reducing their efficacy and requiring careful optimization of extraction parameters.

For the efficient optimization of extraction parameters for bioactive compounds from plants, response surface methods (RSM) are a valuable tool. In this context, RSM has been employed in a limited number of studies involving UAE, often utilizing a range of solvents like water, methanol, ethanol, and others for the extraction process. While certain solvents may offer improved extraction efficiency, they can also pose health risks and toxicity concerns. Considering factors such as cost, health implications, and safety, water stands out as the most suitable solvent. RSM strategies offer advantages in terms of time and labor efficiency compared to other approaches (Chakraborty, Uppaluri, & Das, 2020). This is primarily due to the reduced number of experimental trials required to analyze numerous parameters and their interactions. Many studies have employed widely used RSM designs, such as the central composite design (CCD) and the Box-Behnken Design (BBD). The BBD, primarily used in most RSM investigations, is specifically developed for fitting second-order models and is notable for requiring only three levels per factor to accommodate a second-order regression model, resulting in a reduced number of experiments compared to the CCD, which requires five levels per factor. Therefore, the objective of this study was to assess the physicochemical and color properties of butterfly pea and employ RSM for the optimization of UAE parameters (ultrasonic time, solid-to-solvent ratio, and ethanol concentration) for determining the yield, TPC, TAC and antioxidant activity of butterfly pea flower extract using the BBD.

## 2 Materials and methods

### 2.1 Materials preparation

BPFs were sourced from the local market in Bang Sean, Chonburi, Thailand, in July 2023, at coordinates latitude of 13.2688°N and longitude of 100.9274°E. BPFs were hand-harvested after approximately 45 days of growth, preferably in the early morning, before noon. BPFs were promptly subjected to drying in a drying oven (Model: UM500, Memmert, Germany) at 50 °C for 18 h, resulting in a moisture content reduction to around 15% (wet basis). The desiccated BPF sample were subsequently gathered and transformed into a powdered form utilizing a mixer grinder at speed of 32,000 rpm (Model: YB-800 A, Yun Bang, China). Subsequently, the BPF powder underwent sieving with a laboratory sieve, with all particles passing through an 80-mesh sieve being collected in a polypropylene plastic bag for subsequent analysis.

### 2.2 BPF extraction by UAE

The UAE process utilized an ultrasonic bath (Tru-sweep 950D, Crest, USA) with a 2.5 L capacity, operating at 250 watts of power and a frequency of 45 kHz. BPF powder was prepared into a beaker size 100 mL and used for each experiment according to the Box-Behnken experimental design (Tables 1), which follows a three-level, three-factor full factorial design with center points. The three independent variables included the sample-to-solvent ratio: g:mL (X_1_), ultrasonic extraction time: time (X_2_), and ethanol concentration: % (X_3_). Throughout the extraction procedure, the initial temperature was set at room temperature (28 °C). To prevent the temperature from surpassing 35 °C, meticulous control was exercised by modulating the water level within the ultrasonic bath. This adjustment ensured that the temperature did not exceed 35 °C. Upon completion of the extraction process, the sample solution was centrifuged at 8000 *g* for 15 min using a Hermle centrifuges (HERMLE Labortechnik, Germany) and then the supernatant was filtered through Whatman filter paper (no. 4). The filtered samples were stored in amber flasks at 4 °C, sealed, and capped for subsequent analyses.

**Table 1:** BBD of three variables with their observed responses of BPF extracts.

**Run**⁎	**Independent variables**			**EY** **(%)**	**TPC** **(mg GAE/ g dried extract)**	**TAC** **(mg/100 g dried extract)**	**DPPH** **(**μmole **TE/mg dried extract)**
	**X**_**1**_ **(g:mL)**	**X**_**2**_ **(min)**	**X**_**3**_ **(%)**				
1	20	30	60	41.8 ± 0.72	7.81 ± 0.08	55.91 ± 2.48	87.18 ± 3.42
2	40	30	60	44.55 ± 0.49	13.51 ± 0.23	56.27 ± 2.68	113.59 ± 7.51
3	20	60	60	45.47 ± 0.23	7.23 ± 0.28	42.32 ± 0.47	82.55 ± 5.66
4	40	60	60	48.89 ± 0.11	10.81 ± 0.24	51.73 ± 2.13	106.2 ± 3.30
5	20	45	40	46.32 ± 1.71	7.17 ± 1.26	51.65 ± 2.25	92.36 ± 8.51
6	40	45	40	50.2 ± 0.45	9.52 ± 0.72	50 ± 2.32	107.33 ± 4.34
7	20	45	80	37.71 ± 0.69	7.48 ± 0.33	39 ± 1.13	93.32 ± 5.99
8	40	45	80	41.13 ± 0.65	13.9 ± 0.58	39.95 ± 0.98	131.81 ± 1.21
9	30	30	40	44.83 ± 0.94	8.43 ± 0.89	51.53 ± 2.36	109.11 ± 4.38
10	30	60	40	46.7 ± 0.78	8.16 ± 0.44	44.56 ± 1.81	114.54 ± 2.28
11	30	30	80	41.44 ± 3.35	13.12 ± 3.94	35.73 ± 0.86	112.52 ± 7.69
12	30	60	80	40.55 ± 1.71	9.19 ± 1.2	38.77 ± 0.88	124.67 ± 6.13
13	30	45	60	43.39 ± 0.94	9.52 ± 0.36	54.52 ± 2.92	111.59 ± 1.81
14	30	45	60	42.67 ± 0.95	9.31 ± 0.8	53.28 ± 2.16	128.6 ± 2.01
15	30	45	60	42.04 ± 0.74	9.11 ± 0.71	51.78 ± 1.39	118.31 ± 2.35

⁎ Randomized experiments were conducted.

### 2.3 Experimental design and optimization

In this study, a BBD with three levels and three factors was employed to assess the impact of process variables related to UAE on response variables. Three specific extraction process variables, denoted as X_1_ (solid-to-liquid ratio, ranging from 1:20 to 1:40 (g:mL)), X_2_ (ultrasonic extraction time, between 30 and 60 min), and X_3_ (ethanol concentration, varying from 40% to 80%), were selected. A total of 15 experimental runs were conducted randomly as outlined in Table 1. The data collected during these experiments were used to develop a comprehensive second-order polynomial model, yielding the regression coefficients presented in Eq. (1).

(1) $$Y=\beta_{0}+\sum_{i=1}^{4}\beta_{i}X_{i}+\sum_{i=1}^{4}\beta_{\mathrm{ii}}X_{i}^{2}+\sum_{i=1}^{3}\sum_{j=i+1}^{4}\beta_{\mathrm{ij}}X_{i}X_{j}$$

In this equation, *Y* represents the response function, where *β*_0_ is the intercept, and *β*_i_, *β*_ii_, and *β*_ij_ are the coefficients for the linear, quadratic, and interactive terms, respectively. The variables *X*_i_, *X*_i_^2^, and *X*_i_*X*_j_ represent the coded independent variables, respectively.

The analysis of variance (ANOVA) was performed using Minitab version 18 software to determine the significance of interactions within the model (*p* < 0.05). Statistical significance was confirmed by an F-test. The fitness of the polynomial model equation was verified by analyzing the lack of fit using an F-test. Results with p < 0.05 were considered significant, and they were checked against the lack of fit. Coefficient of determination R^2^, adjusted R^2^, and adequate precision were also examined for model suitability. Graphical and numerical optimization techniques were employed to determine optimal independent variable levels. Confirmation experiments were then conducted to validate the optimal conditions.

### 2.4 Analysis of extraction yield (EY)

To determine the extraction yield, the solvent was removed by evaporation, and the remaining sample was considered as the yield. Briefly, 50 mL of the BPF extract were transferred to a stainless-steel moisture container and subsequently placed into a hot air oven (UM500, Memmert, Germany) set at 105 °C for 18 h. After the drying process, the dried samples were weight and calculated using the Eq. (2).

(2) $$\text{Extraction yield}=\left(\text{weight obtained after extraction}/\text{initial weight}\right)\;x\;100$$

### 2.5 Analysis of total anthocyanin content (TAC)

The total anthocyanin content is determined as follows, with modifications based on Lee, Durst, and Wrolstad (2005). BPF extract was diluted to an appropriate concentration using distilled water. Each sample was divided into two set, one being mixed with 0.2 M KCl, pH 1.0, and the other with 0.2 M acetate buffer (pH 4.5). Subsequently, these mixtures were incubated at room temperature for a duration of 5 min to facilitate equilibrium attainment. The absorbance measurements were then performed at 510 and 700 nm using disposable cuvettes with a 1 cm path length on a UV–Vis spectrophotometer (10S UV–Vis Spectrophotometer, Genesys, USA), while instrumental zero calibration was achieved using distilled water. The ΔAbsorbance values were employed to estimate the total anthocyanin concentration in the sample through the application of Beer's Law. The equation is as follows Eq. (3):

(3) Total anthocyanin=ΔAbsorbance×Molar absorptivity×Dilution factor/ε×l

where ΔAbsorbance is the absorbance differences for each sample by subtracting the absorbance at the higher pH from the absorbance at the lower pH. Molar absorptivity is a constant for anthocyanins at the given wavelength. ε is molar extinction coefficient of cyanidin-3-glucoside (26,900 M^−1^ cm^−1^). *l* represents the cell's path length, typically set at 1 cm.

### 2.6 Analysis of total phenolic compounds (TPC)

The analysis of TPC was determined following the Folin-Ciocalteu method (Swain & Hillis, 1959). 50 μL of the extract, 200 μL of de-ionized water, and 50 μL of Folin-Ciocalteu reagent were combined in a quartz vial and thoroughly mixed using a Vortex. After allowing the mixture to react for 5 min, 500 μL of a 7% (*w*/*v*) Na_2_CO_3_ solution was added and mixed well. The solution was then incubated at room temperature in the dark for 60 min. Subsequently, the absorbance was measured at 760 nm using a UV-spectrophotometer (10S UV–Vis Spectrophotometer, Genesys, USA), and the results were expressed in gallic acid equivalents (mg GAE/100 g dry sample) utilizing a gallic acid (0–50 mg/mL) standard curve. In cases where the absorbance value exceeded the linear range of the standard curve, additional dilution was performed.

### 2.7 Analysis of antioxidant activity using the DPPH method

The assessment of antioxidant activity was performed by employing the DPPH method, with modifications following Brand-Williams et al. (1995). A volume of 500 μL of BPE extracts, diluted one hundred times with distilled water, is combined with an equivalent volume of 500 μL of 0.5 mM DPPH solution (in 95% ethanol). The reaction solution was adequately mixed with a Vortex machine and kept in the dark for 30 min. Subsequently, absorbance was measured at a wavelength of 517 nm using a UV-spectrophotometer. A standard curve was established using Trolox, and the results are reported in Trolox equivalent antioxidant capacity (μmole TE/ mg dried extract).

### 2.8 Impact of pH on the color change of BPF extract

The color response of the BPF extract to pH changes in aqueous media (pH 2–12) was determined. pH buffer solutions (concentration of 0.1 M) at pH 2, pH 4, pH 6, pH 8, pH 10, and pH 12 were prepared using HCl/KCl buffer, KHP (potassium hydrogen phthalate) /HCl buffer, KH₂PO₄/NaOH buffer, Glycine/NaOH buffer, and KCl/NaOH buffer, respectively. BPE was diluted one hundred times with distilled water and dispensed into test tubes. Buffer solutions at different pH were added into the test tubes to observe the color change in response to variations pH. Spectra of BPF extract at different pH were measured using the absorbance values utilizing a spectrophotometer in the wavelength range of 300 to 700 nm.

### 2.9 Color changes of BPF extract during storage

In this study, samples obtained under optimized conditions were concentrated using a rotary evaporator (R-100, BUCHI, Switzerland) at 45 °C and a pressure of 120 millibars. The obtained BPF extract (with the concentration of 20 mg dried extract/mL) stored in amber bottles was incubated at 35 °C for 0, 1, and 2 weeks. Subsequently, the BPE extracts were subjected to color measurement using a Hunter Lab Colorimeter (MiniScan XE Plus, HunterLab, USA). The colorimeter equipment was calibrated using standard white and black tiles or other reference samples. The BPF extract solution was transferred into the sample holder cup, which was then placed within the measurement area, covering the entire measurement surface. The recorded color values are expressed in the CIE LAB system including L* (lightness), a* (red-green), and b* value (yellow-blue).

### 2.10 Impact of BPF extract concentration in coconut milk jelly on the color properties

The investigation focused on the impact of varying concentrations of obtained BPF extract in coconut milk jelly (a Thai dessert) on color appearance. In summary, the preparation involved combining 45 g of agar powder with 300 mL of water, stirring thoroughly, and letting it rest for 10–15 min before heating. Coconut water (300 mL) was then added, and the mixture was heated over medium heat with constant stirring until the agar powder dissolved. Sugar (280 mL) and salt (5 g) were incorporated, ensuring complete dissolution. After bringing the mixture to a boil, the heat was reduced to low, and coconut milk was added while stirring continuously for 2 min. BPF extract was then added at different concentrations (0.5%, 1%, 2%, and 5%) for each treatment. The ultimate pH of the mixed solution was 6.5. The solution was transferred into flower-shaped molds, left to set in the refrigerator (4 °C) for 1 h, and then demolded for subsequent color analysis using a Hunter Lab Colorimeter.

### 2.11 Statistical analysis

The experiment was conducted in triplicate. Data and variances were analyzed using ANOVA, and means were compared using Tukey's test at a 95% confidence level. Correlation analysis was performed using the Pearson correlation method. All analyses were conducted using Minitab 18 statistical software.

## 3 Results and discussion

### 3.1 Regression and variance analysis (ANOVA)

The RSM technique, utilizing a Box-Behnken design, was employed to conduct a 3-factor, 3-level experiment for the optimization of colorants derived from butterfly pea flowers through UAE process. A comprehensive set of 15 experimental designs is detailed in Table 1. A regression model was utilized to establish the relationship between the independent variables (extraction parameters) and the dependent variables (EY, TPC, DPPH, and TAC). The results of the experiment demonstrated a range of outcomes: the total yield ranged from 37.71 to 50.20 mg/100 g, while the total phenolic compound content varied between 7.17 and 13.90 mg GAE/g sample. Additionally, the anthocyanin values spanned from 35.73 to 56.27 mg/100 g sample, and the antioxidant activity, assessed by the DPPH method, ranged from 82.55 to 131.81 μmol TE/mg sample, as detailed in Table 1. This comprehensive analysis provides valuable insights into the various parameters governing the UAE process.

The ANOVA suggested that quadratic, second-order polynomial models were the most suitable choice for effectively describing the responses during the experimental procedures in the UAE process. The derivation of predictive quadratic models involved the use of a backward elimination regression approach. Statistically non-significant terms were eliminated, with the retention of those deemed essential to maintain the hierarchical structure. The factors ranged from −1 to +1, and their impact on the response variables was evaluated using correlation coefficients (R^2^) with statistical significance at *p* < 0.05. The relationship between the factors and responses was considered significant (p < 0.05) based on the quadratic polynomial model. Furthermore, the lack of fit test yielded statistically non-significant results (*p* > 0.05), as indicated by the calculated F values for the EY, TPC, TAC, and DPPH activity, respectively. Additionally, small pure error values were observed, signifying the data's good repeatability and a satisfactory coefficient of determination. The analysis of the relationship between the studied factors and the response values revealed a regression equation model with significance at *p* < 0.05. Table 2 displays the effects of all the examined parameters, their corresponding F-values, and the associated probability (*p*-value). These coefficients were computed using the coded values of the parameters. The correlation coefficient of the EY reached 0.8997, while the adjusted R^2^ value was 0.7192. This suggests that the model can predict the EY closely in accordance with the actual experimental results (*p* < 0.05). The analysis revealed that X_1_ and X_3_ had a significant linear influence on the EY (p < 0.05) as indicated by F-values of 7.30 (p -value = 0.043). Conversely, X_2_ did not exert a significant linear influence on the EY (*p* > 0.05). The results were fitted using multiple regression, and the relationship between the EY of BPF and the variables was expressed by the following Eq. (4):

(4) $$\mathrm{EY}=57.66–0.68{\text{4X}}_{1}–0.22{\text{4X}}_{2}+0.0749X_{3}+0.0142{X_{1}}^{2}$$

**Table 2:** Variance analysis for the fitted models (EY and TPC).

**Source**	**DF**	**EY**				**TPC**			
		**Adj SS**	**Adj MS**	**F**	***p*-value**	**Adj SS**	**Adj MS**	**F**	**p-value**
Model	9	139.368	15.4854	4.98	0.046*	70.4406	7.8267	110.24	0.000*
X_1_	1	22.680	22.6801	7.30	0.043*	40.7253	40.7253	573.61	0.000*
X_2_	1	10.103	10.1025	3.25	0.131	6.9938	6.9938	98.51	0.000*
X_3_	1	92.616	92.6161	29.81	0.003*	13.5460	13.5460	190.79	0.000*
X_1_^2^	1	7.965	7.9651	2.56	0.170	0.0940	0.0940	1.32	0.302
X_2_^2^	1	3.757	3.7572	1.21	0.322	0.4975	0.4975	7.01	0.046*
X_3_^2^	1	0.399	0.3991	0.13	0.735	0.0073	0.0073	0.10	0.761
X_1_X_2_	1	0.112	0.1122	0.04	0.857	1.1236	1.1236	15.83	0.011*
X_1_X_3_	1	0.053	0.0529	0.02	0.901	4.1412	4.1412	58.33	0.001*
X_2_X_3_	1	1.904	1.9044	0.61	0.469	3.3489	3.3489	47.17	0.001*
Error	5	15.532	3.1065			0.3550	0.0710		
Lack-of-fit	3	14.620	4.8732	10.68	0.087	0.2709	0.0903	2.15	0.333
Pure error	2	0.913	0.4563			0.0841	0.0420		
Total	14	154.901				70.7956			
R^2^		89.97%				99.50%			
R^2^(adj)		71.92%				98.60%			

DF: degrees of freedom; SS: sum of squares; MS: mean square; * stand for significant differences (p < 0.05).

In the analysis of TAC, the model exhibited a relative value of 0.9375 and an adjusted R^2^ value of 0.8249 (Table 3). It was observed that X_2_ had a significant linear influence on TAC (*p* < 0.05) with an F-value of 7.11 and a P -value of 0.045. The linear effect of ethanol concentration (X_3_) on TAC was significant, as well as the quadratic effect of ultrasound extraction time on TPC. When considering the squared term relationship, it was evident that X_3_ significantly affected TAC (*p* < 0.05) as a single factor. Nevertheless, when examining the interaction effect of two factors (2-way interaction), no significant interaction was observed between the factors (*p* > 0.05). Consequently, an equation to predict the TAC of BPFs was derived by the following Eq. (5):

(5) $$\mathrm{TAC}=1.9–0.1838X_{2}+2.23{\text{1X}}_{3}+0.0043{X_{1}}^{2}–0.0209{X_{3}}^{2}$$

**Table 3:** Variance analysis for the fitted models (TAC and DPPH).

**Source**	**DF**	**TAC**				**DPPH**			
		**Adj SS**	**Adj MS**	**F**	**p-value**	**Adj SS**	**Adj MS**	**F**	**p-value**
Model	9	641.673	71.297	8.33	0.016*	2741.07	304.56	5.53	0.037*
X_1_	1	10.260	10.260	1.20	0.324	1339.55	1339.55	24.31	0.004*
X_2_	1	60.830	60.830	7.11	0.045*	3.86	3.86	0.07	0.802
X_3_	1	245.311	245.311	28.65	0.003*	189.93	189.93	3.45	0.122
X_1_^2^	1	0.693	0.693	0.08	0.787	894.25	894.25	16.23	0.010*
X_2_^2^	1	15.847	15.847	1.85	0.232	158.77	158.77	2.88	0.150
X_3_^2^	1	265.307	265.307	30.99	0.003*	18.98	18.98	0.34	0.583
X_1_X_2_	1	20.430	20.430	2.39	0.183	1.90	1.90	0.03	0.860
X_1_X_3_	1	1.690	1.690	0.20	0.675	138.30	138.30	2.51	0.174
X_2_X_3_	1	25.100	25.100	2.93	0.148	11.29	11.29	0.20	0.670
Error	5	42.805	8.561			275.46	55.09		
Lack-of-fit	3	39.026	13.009	6.88	0.129	128.67	42.89	0.58	0.681
Pure error	2	3.779	1.890			146.79	73.40		
Total	14	684.478				3016.53			
R^2^		93.75%				90.87%			
R^2^(adj)		82.49%				74.43%			

DF: degrees of freedom; SS: sum of squares; MS: mean square; * stand for significant differences (p < 0.05).

The linear and interaction terms of X_1_, X_2_, and X_3_ were significant (p < 0.05) for TPC. The TPC demonstrated exceptionally high R^2^ and adjusted R^2^ values, indicating a strong correlation with the model (R^2^ = 0.995, adjusted R^2^ = 0.986). In contrast, only the linear and quadratic terms of X1 were significant (p < 0.05) for DPPH. The relationships between the responses (TPC and DPPH) and variables were interpreted through the subsequent second-order polynomial Eq. (6), (7), respectively.

(6) $$\mathrm{TPC}=0.89+0.0794X_{1}+0.0857X_{2}+0.0497X_{3}+0.001567{X_{2}}^{2}–0.003533\;X_{1}X_{2}+0.005087\;X_{1}X_{3-}0.00305\;X_{2}X_{3}$$

(7) $$\text{DPPH}=-83.9+8.97X_{1}+2.74X_{2}–0.63{\text{8X}}_{3}–0.1574{X_{1}}^{2}–0.0299{X_{2}}^{2}+0.0294\;X_{1}X_{3}$$

Consistent with our findings, Koraqi, Petkoska, Khalid, Kumar, and Pareek (2023) also observed an increase in TPC as the ultrasound-assisted extraction time increased, suggesting a positive quadratic relationship. The solvent volume was identified as the second most influential factor positively affecting TPC after the extraction time. In a study on ultrasound-assisted extraction from *Saussurea lappa*, longer extraction times were associated with a negative effect on antioxidant capacity, as indicated by a coefficient of −8.74, suggesting that extended extraction times may not enhance, and could potentially reduce, antioxidant capacity (Ali & Ahmed, 2023).

### 3.2 The effect of various UAE conditions on the EY

**Fig. 1:**
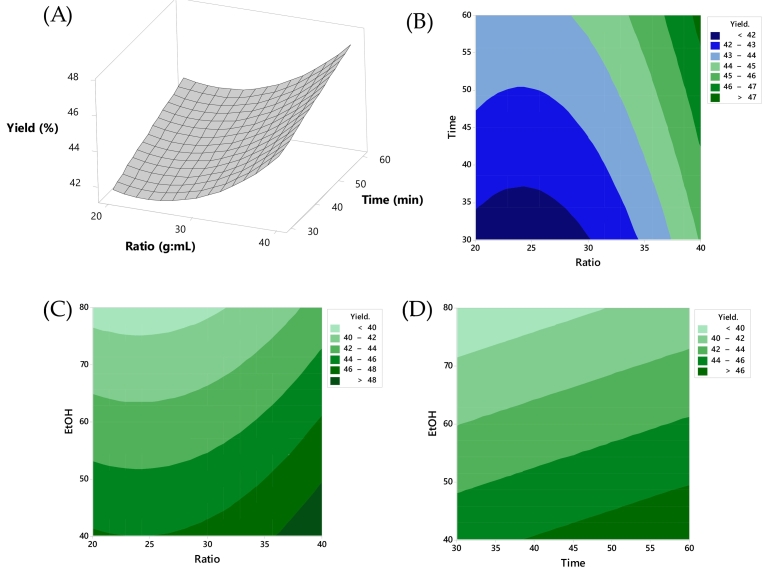
Response surface plots of EY as affected: (A) time and ratio; contour plot between (B) time and ratio, (C) ethanol concentration and solid-to-liquid ratio, and (D) time and ethanol concentration, respectively.

The effect of various components on the EY is illustrated in Fig. 1. It was evident that *X*_1_ (sample-to-solvent ratio) and X_2_ (extraction time) exhibit an increasing trend in yield when the ratio was 40 (*w*/*v*) and the extraction time was 60 min. This observation aligns with Fig. 1(B), which demonstrates that higher X_1_ (1:40) and a longer extraction time of 60 min significantly increased the EY (*p* < 0.05). This effect can be attributed to the increase in the temperature in the solution. During the UAE process, the application of ultrasonic waves generates cavitation bubbles in the liquid medium. The rapid formation and collapse of these bubbles produce localized high temperatures and pressures, leading to an overall increase in the temperature of the solution. This elevated temperature enhances the mass transfer and solubilization of the target compounds, thereby improving the extraction efficiency. Fig. 1(C) indicates that a higher concentration of X_1_ and a lower concentration of X_3_ had a significant influence on the yield. This was supported by the respective *p*-values of X_1_ (0.043) and X_3_ (0.003), indicating an interaction effect between variables X_1_ and X_3_. This finding was consistent with previous research by Baskaran, Mudalib, and Izirwan (2019), which reported that the extraction yield of anthocyanins from BPFs increased with greater solvent volume. Fig. 1(D) reveals that X_2_ (extraction time) with a longer duration and X_3_ with a lower concentration result in increased yield. This outcome was due to the beneficial effects of high-frequency sound waves in extracting phytochemicals, which were biologically active compounds. These sound waves facilitated mass movement and improved solvent penetration into the plant materials used for extraction, thereby enhancing extraction efficiency. In the case of plant materials, the application of high-frequency sound waves promoted swelling, breaking cell walls, and releasing substances in greater quantities. Normally, BPF extracts, rich in anthocyanins and carotenoids, influence food acceptability and properties. Anthocyanins, responsible for the flower's blue color, stabilize color and offer antioxidants (Vidana Gamage, Lim, & Choo, 2021). BPF extracts, abundant in anthocyanins and carotenoids, impact food qualities. Anthocyanins stabilize color and provide antioxidants, while carotenoids contribute yellow-orange hues and antioxidant benefits. Additionally, *C. ternatea* contains various bioactive compounds like alkaloids, tannins, glycosides, and flavonoids, alongside essential minerals such as calcium, magnesium, potassium, and zinc, as well as fatty acids like palmitic acid, stearic acid, petroselinic acid, and linoleic acid (Multisona, Shirodkar, Arnold, & Gramza Michalowska, 2023). Moreover, BPFs contained polysaccharides that were more soluble in water than in ethanol, potentially leading to the extraction of non-pigment substances and increasing the overall product weight (Soria & Villamiel, 2010).

### 3.3 The effect of various UAE conditions on the TAC

**Fig. 2:**
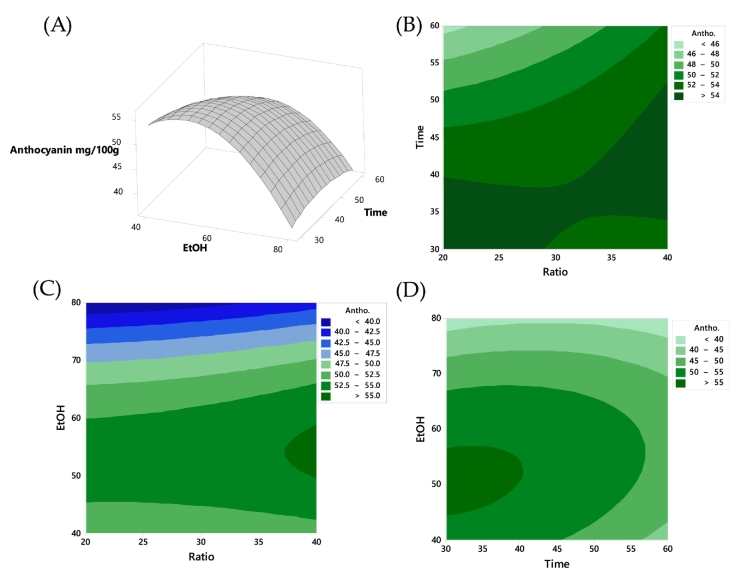
Response surface plots of TAC as affected: (A) time and ratio; contour plot between (B) time and ratio, (C) ethanol concentration and solid-to-liquid ratio, and (D) time and ethanol concentration, respectively.

In Fig. 2(A), as ethanol concentration increases, anthocyanin content decreases, suggesting an inverse relationship, consistent with Fig. 2(D) where extraction time (X_2_) should not exceed 40 min. The interaction effect between the variables is further demonstrated in Fig. 2(B), which presents a contour plot illustrating the influence of X_1_ and X_2_. It was evident that higher anthocyanin content was achieved with a combination of a short extraction time and a greater solid-to-solvent ratio. Fig. 2(C) displays a contour plot highlighting the influence of X_1_. According to the findings of Zhang, Fan, Khan, Yan, and Beta (2020), extremely short or excessively long extraction times did not yield high levels of anthocyanin. In cases of too short an UAE time, anthocyanins might not fully dissolve in the extraction solvent. Conversely, extended extraction at high temperatures could lead to the degradation of anthocyanins, as these compounds were temperature sensitive. Additionally, as reported by Li, Zhang, Wang, Feng and Han (2023), ethanol was more effective in extracting anthocyanins than deionized water. The relationship between ethanol concentration and the extraction rate of anthocyanins was not linear. According to Fig. 2, the extraction rate of anthocyanins was highest within a certain range of ethanol concentrations, rather than continuously increasing with higher ethanol concentrations. This phenomenon can be attributed to the dual role of ethanol as both a solvent and an extraction enhancer. At optimal ethanol concentrations, the solvent polarity is balanced, which effectively solubilizes anthocyanins and maximizes their extraction. However, beyond this optimal range, further increases in ethanol concentration may reduce the solvent's ability to interact with water-soluble anthocyanins, thus decreasing the extraction efficiency. Therefore, the highest extraction rate was achieved within a specific ethanol concentration range where the solvent properties are most favorable for anthocyanin solubilization. Goula, Ververi, Adamopoulou, and Kaderides (2017) found that solid-to-liquid ratio of 100 g/L was optimal for anthocyanin extraction from wine lees, with a slight decrease as the ratio decreased to 25 g/L. More and Arya (2021) as well as Chen, Yang, Mou, and Kong (2018) studied this factor within the ranges of 20–100 g/L and 28.6–66.7 g/L, respectively. The consensus from these studies is that increasing the solid-to-liquid ratio decreases the extraction yield, aligning with mass transfer phenomena expectations, where a higher gradient enhances the driving force for mass transfer. However, lower solid-to-liquid ratios may lead to increased solvent consumption, necessitating investigation for each specific case to determine the optimum ratio. The impact of ultrasound power intensity on anthocyanin extraction is noteworthy; for instance, increasing ultrasonic power up to 300 W maximized anthocyanin content from mulberry wine residues, but further increases reduced yield (Zhang et al., 2020). Similar trends were observed in anthocyanin extraction from jabuticaba peel, where increasing ultrasound intensity up to 7.3 W/cm^2^ increased anthocyanins, but at 13 W/cm^2^, the yield decreased (Gadioli Tarone et al., 2021). However, power level variations may not consistently affect pigment extraction, as observed in the ultrasonic-assisted extraction of anthocyanins from grape pomace (da Rocha & Noreña, 2020). Concerning processing time, samples with higher concentration may require longer extraction times for effective cell disruption and increased pigment release, as demonstrated in anthocyanin extraction from eggplant peel cut into squares (Ferarsa et al., 2018). Conversely, excessively long extraction times can lead to reduced yield (Zafra-Rojas et al., 2020). The discussion emphasizes the importance of ultrasonic power and amplitude for the extraction of compounds from plants. However, in this experiment, the ultrasonic power and amplitude were kept constant and were not included as independent variables in the Box-Behnken design. While ultrasonic power and amplitude are indeed critical factors influencing extraction efficiency, the focus of this study was to optimize the conditions related to solvent concentration, extraction time, and material-to-solvent ratio. Future studies could incorporate ultrasonic power and amplitude as variables to further refine and optimize the extraction process.

### 3.4 The effect of various UAE conditions on the TPC

**Fig. 3:**
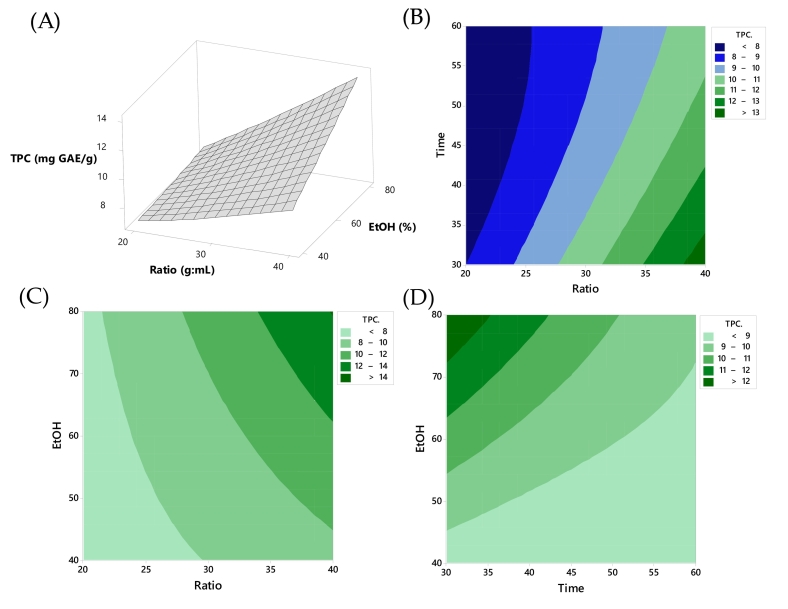
Response surface plots of TPC as affected: (A) time and ratio; contour plot between (B) time and ratio, (C) ethanol concentration and solid-to-liquid ratio, and (D) time and ethanol concentration, respectively.

Fig. 3 illustrates the influence of extraction conditions on TPC. Higher solid-to-solvent ratio correlated with increased phenolic content, supported by Fig. 3(C) where high solid-to-solvent ratio combined with elevated ethanol concentration results in greater phenolic content. Fig. 3(B) presents a contour plot revealing the influence of X_1_ and X_2_ (extraction time). It indicated that a high X_1_ and a low X_2_ led to higher phenolic compound content. Conversely, Fig. 3(D), a contour plot depicting the influence of X_2_ and X_3_, suggests that a low X_2_ (short extraction time) and a high X_3_ (ethanol concentration) resulted in increased phenolic compound content. These observations aligned with the findings of Leong, Juliano, and Knoerzer (2017), who noted that increasing the amplitude enhances the efficiency of breaking cell walls, facilitating the release of important compounds into the solvent. However, an extended extraction time might lead to decreased extraction efficiency due to two distinct mechanisms. Initially, during the early stages of extraction, solutes on the surface of the plant material were dissolved, which could be further enhanced with increased extraction time. Subsequently, a slower extraction process was initiated, involving a gradual release of bioactive compounds from the plant matrix into the solvent. In the RSM plot of Fig. 3(C), the TPC exhibited the highest value, reaching 14 mg GAE/g sample at the condition of ethanol concentration of 80% and the ratio of 1:40 (g:mL). This is consistent with other studies that demonstrated the significant impact of ethanol concentration on phenolic compound extraction. For instance, *citrus limon* fruit extracted with 63.93% ethanol yielded 1502.2 ± 0.88 mg GAE/100 g (Dahmoune et al., 2013), while *Pistacia lentiscus* leaves obtained a lower TPC value of 142.76 ± 19.98 mg GAE/g when using 46% ethanol for extraction. (Dahmoune et al., 2014). Additionally, Esmaeelian, Jahani, Feizy, and Einafshar (2020) reported a lower TPC value from saffron corms extract (100.39 mg GAE/100 g dry saffron corm) using 80% ethanol compared to our study (900–1300 mg GAE/100 g). The results of this experiment demonstrated that the use of ultrasonic waves aided in achieving good extraction efficiency. Ultrasonic waves generate microjets, causing disruptions in the cell walls, a phenomenon known as cavitation, which accelerated the mass transfer rate and facilitates the release of plant compounds into the solvent. Arruda et al. (2019) investigated the impact of high-intensity ultrasound parameters, including nominal power (160–640 W) and extraction time (0.5–5.0 min), on the recovery of phenolic compounds from araticum peel. The study revealed a significant influence of these process parameters on phenolic recovery, indicating that ultrasound-assisted extraction disrupts plant cell walls through cavitation phenomena.

### 3.5 The effect of various UAE conditions on the DPPH

**Fig. 4:**
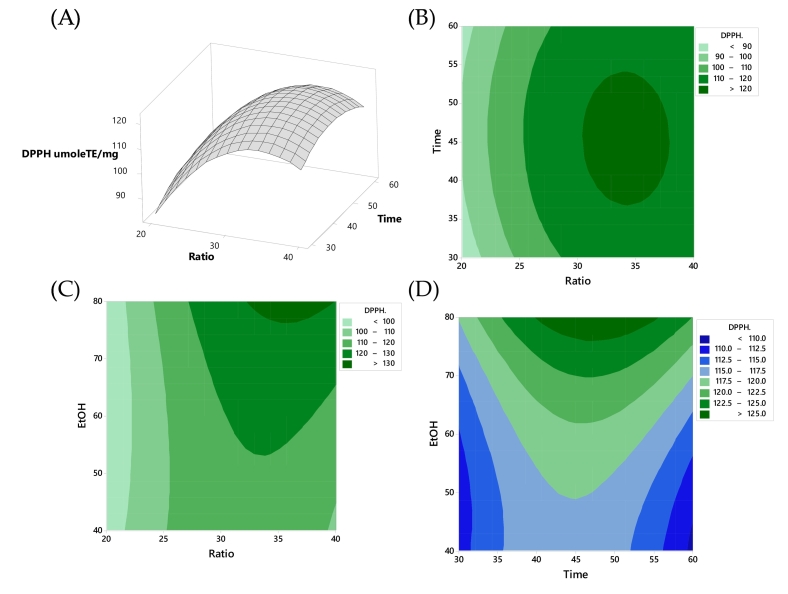
Response surface plots of DPPH as affected: (A) time and ratio; contour plot between (B) time and ratio, (C) ethanol concentration and solid-to-liquid ratio, and (D) time and ethanol concentration, respectively.

The impact of extraction conditions on antioxidant activity, assessed through the DPPH method, is depicted in Fig. 4. With the increase of the solid-liquid ratio, the DPPH scavenging ability initially rises and then decreases. This indicates that there was an optimal solid-liquid ratio where the DPPH scavenging activity was maximized, rather than a linear increase with higher solid-liquid ratios. This behavior suggests that beyond a certain point, the extraction efficiency may be hindered by too much solid material, leading to decreased DPPH scavenging ability. The contour plot in Fig. 4(B) suggests that peak antioxidant activity was achieved with higher values of both solid-to-solvent ratio and time, approximately within the range of 45–50 min. Fig. 4(C) presents a contour plot illustrating that heightened X_1_ and increased concentrations of X_3_ (ethanol concentration) contribute to elevated antioxidant activity. Additionally, Fig. 4(D) indicates that a shorter extraction time (low X_2_) coupled with a high X_3_ concentration led to increase the antioxidant activity. These findings are in line with the research of Berkani et al. (2020), which reported different solvent concentrations resulting in various polarities led to varying solubilities of the substance. Water serves as an efficient solvent for extracting polar molecules, while for less polar compounds, greater efficiency can be achieved with ethanol. An increase in ethanol concentration significantly reduced the solvent's polarity. The decrease in solvent polarity with increasing ethanol concentration led to reduced extraction of substances from the plant material. This was because less polar solvents may not efficiently extract certain compounds compared to more polar solvents. Additionally, the high antioxidant activity of the extracted anthocyanins could be attributed to their hydroxyl-rich structure, particularly on the B-ring, which enables effective free radical scavenging (Kongpichitchoke, Hsu, & Huang, 2015). This structural configuration was responsible for the heightened antioxidant effects observed in materials rich in anthocyanins and phenolic compounds. It had been documented in previous studies that such compounds exhibit elevated antioxidant potential due to their high number of hydroxyl groups (Benavente-García, Castillo, Lorente, Ortuño, & Del Rio, 2000; Kongpichitchoke et al., 2015). The inhibition of DPPH was linked to the abundance of phenolic compounds (Berkani et al., 2020). Additionally, other compounds like ascorbic acid, tocopherol, and pigments may contribute synergistically to overall antioxidant activity, indicating a role beyond phenolic compounds alone (Benhanifia, Mohamed, Bellik, & Benbarek, 2013). Ultrasound extraction proved more efficient in obtaining antioxidant-rich polyphenols from BPF. The enhanced recovery and antioxidant activity were attributed to ultrasound's mechanical cavitation.

### 3.6 Optimization and validation

Utilizing the response surface model, we employed numerical optimization techniques to determine the optimal UAE conditions for colorants from BPF. The objective was to identify the combination of variables that would yield the highest predicted value in all response (EY, TAC, TPC, and DPPH). The optimal conditions for independent variables and the predicted values of the response were determined as follows: a solid-to-solvent ratio of 1:40 (g/mL), an ethanol concentration of 37% (*v*/v), and an extraction time of 61 min. As shown in Table 4, the optimized condition predicts the following values: EY = 45.29%, TAC = 54.09 mg/100 g sample, TPC = 12.76 mg GAE/g sample, and DPPH = 115.5 μmole TE/mg sample. In the pursuit of finding the ideal conditions, all three variables were taken into consideration. The combined analysis of all four variables yielded desirability values of 0.6688 (EY), 0.89427 (TAC), 0.8307 (TPC), and 0.60693 (DPPH), resulting in an overall suitability value for the model of 0.7411, with a typical range of 0 to 1, where a value closer to 1 indicates a more appropriate model. Our study achieved optimal UAE conditions for extracting phenolics and antioxidants from BPF that showed similar results from the different plant materials. Melgar et al. (2019) who optimized the extraction of bioactive compounds from *Opuntia engelmannii* peel using extraction time (t), solid-to-liquid ratio (S/L), methanol concentration, and temperature (T) reported that the optimum values were achieved at *t* = 2.5 min, S/L = 5 g/L, metOH = 34.6%, and *T* = 30 °C, resulting in maximum responses of 13.9 mg of phenolic acids/g, 2.4 mg of flavonoids/g, and 71.8% of extractable solids. Umair et al. (2021) determined that the optimal conditions for ultrasound-assisted extraction of carotenoids from carrot pomace were 17 min of extraction time, a temperature of 32 °C, and an ethanol concentration of 51%. Additionally, our study's optimized conditions featured higher ethanol concentration and longer extraction time compared to the conditions used by Plazzotta, Ibarz, Manzocco, and Martín-Belloso (2020) in their work on antioxidant compounds from peach waste.

**Table 4:** Predicted and experimental values under the optimal operating conditions.

**Response variable**	**Unit**	**Predicted value**	**Experimental value**⁎
EY	%	45.29	41.01 ± 3.74
TAC	mg/100 g sample	54.09	48.49 ± 2.27
TPC	mg GAE/g sample	12.76	13.68 ± 1.85
DPPH	μmole TE/mg sample	115.5	129.53 ± 1.61

⁎ The value represents the mean of three replications with standard error.

The model verification utilized selected cases based on the optimization results obtained from the response surface model. The confirmation experiments conducted under the optimal conditions predicted by the model to assess the model's accuracy, a comparison was made between the actual experimental value and the predicted value. Implementing the optimized conditions for colorant extraction from BPFs resulted in the EY of 41.01 ± 3.74 g/100 g sample, TAC of 48.49 ± 2.27 mg/100 g sample, TPC of 13.68 ± 1.85 mg GAE/g sample, and DPPH of 129.53 ± 0.161 μmole TE/mg sample. Comparison of the experimental value and the predicted value demonstrated similar results, validating the accuracy of the model. These experimental values closely matched the predicted values. Furthermore, when considering percent error, it was determined to be 9.45, 10.35, 7.21, and 12.15 for EY, TAC, TPC, and DPPH, respectively. Within an acceptable threshold of 10%, validate the reliability of the conditions for colorant extraction from BPFs predicted by the mathematical model, affirming its utility for predictive purposes.

### 3.7 Impact of pH on the color change of BPF extract

**Fig. 5:**
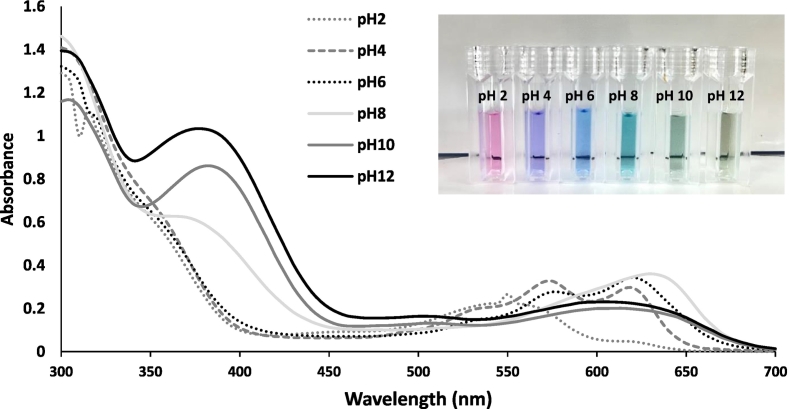
Relationship between the wavelength and the absorbance of the colorant from butterfly pea flowers at pH 2–12.

The study investigated the impact of pH on the color change of BPE extract. The results, as displayed in Fig. 5, revealed significant variations in the extract's color based on the pH levels. At pH 2, the extract showed maximum absorption at 300 nm, absorbing green light and producing a pink color. At pH 4, the highest absorption occurred at 575 nm, indicating strong absorption of yellow light and a purple hue. At pH 6, the λ_max_ was 625 nm, signifying absorption of orange light and a blue color. Between pH 8 and 12, the extract displayed an λmax at 300 nm, absorbing red light and resulting in a green color. In various pH conditions, anthocyanins display a range of colors from red in acidic environments to purple in neutral pH and blue at higher pH levels. The stability of red-colored anthocyanins, existing as flavylium cations, is higher in lower pH solutions. The solubility of anthocyanins increases in lower pH due to the formation of highly soluble flavylium cations. However, as pH rises, the increased deprotonation rate of flavylium cations results in reduced color stability. Anthocyanin-tannin polymerization enhances color stability in lower pH conditions. At higher pH, colorless carbinol pseudobase and chalcone structures form, leading to the generation of anionic quinonoidal species (Fossen, Cabrita, & Andersen, 1998). This occurs due to the kinetic and thermodynamic competition between the hydration reaction of flavylium ions. The blue quinonoidal species is unstable at lower pH, and at pH 4–5, an anthocyanin solution exhibits minimal hue. At neutral pH, resonance-stabilized quinonoid anions, representing the purple color of anthocyanins, form through further deprotonation of the quinonoidal species (Coutinho, Freitas, & Maçanita, 2015). The alteration in the color of BPE extracts across various pH levels is attributed to changes in the structure of anthocyanin pigments, which depend on the number of hydroxyl groups within the molecule (Hasanah, Mohamad Azman, Rozzamri, Zainal Abedin, & Ismail-Fitry, 2023). Anthocyanins are classified as phenolic compounds, characterized by a chromophore with a benzene ring. The groups attached to the chromophore affect the molecule's absorption spectrum, shifting it toward longer wavelengths (Campbell, Pearson, & Marble, 2019). These findings align with previous research that indicated the stability of anthocyanin depends on factors such as pH, with low pH levels enhancing stability, as well as the influence of light (Hasanah et al., 2023). This phenomenon, known as Bathochromic or a red shift, refers to the alteration in the absorption or emission wavelengths of a substance, often resulting in a change in color. This shift can occur when various environmental factors, including pH levels and the type of solvent, change. It is an essential characteristic of certain compounds, including anthocyanins, which are known for their sensitivity to changes in pH and solvent conditions (Wiyantoko, 2020). The pH sensitivity of BPF anthocyanins could prove advantageous for their use in food colorants. Anthocyanins can adjust their color based on pH, making them adaptable to various food products with different acidity levels. This versatility allows for a broad spectrum of colorful options without relying on synthetic additives. Moreover, the natural vibrancy and stability of anthocyanin colors enhance the visual appeal of food, meeting consumer preferences for natural and clean-label ingredients. Thus, BPF anthocyanins offer versatility, visual appeal, and alignment with consumer demands, making them ideal for natural food coloring in diverse applications.

### 3.8 Color changes of BPF extract during storage

**Fig. 6:**
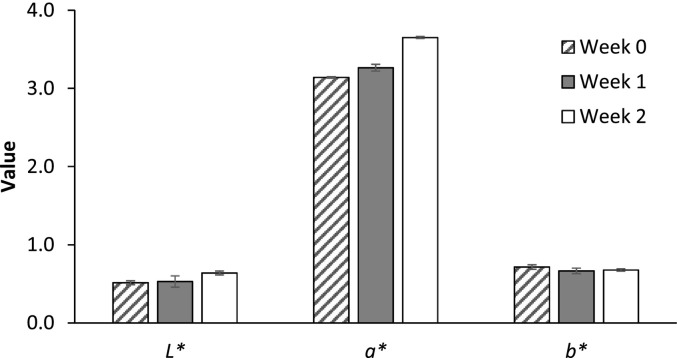
Color changes of BPF extract during storage at 35 °C for 2 weeks.

The color variations observed in the pigment extracted from BPF under optimal UAE conditions, with a concentration of 20 mg dried extract/mL, during storage at 35 °C for 0, 1, and 2 weeks exhibited minor alterations, as illustrated in Fig. 6. The brightness value (L*) slightly increased in the range of 0.51–0.6, indicating a tendency toward enhanced brightness compared to the initial conditions at week 0. The positive a* value, which represents redness, increased within the range of 3.1–3.64 when compared to week 0. Conversely, the b* value, signifying yellow color, was positive but displayed a decrease within the range of 0.68–0.71. These results are consistent with research by Charurungsipong, Tangduangdee, Amornraksa, Asavasanti, and Lin (2020), who found that a decrease in anthocyanin content with prolonged heating time, and concurrently, higher temperatures were associated with lower anthocyanin content. The anthocyanin stability depends on several factors, including the structure of the molecule, the presence and number of sugar molecules near the flavylium ion, the number of glycosidic acid linkages, and the number and position of substitutions on the flavylium ion (Khoo, Azlan, Tang, & Lim, 2017). For instance, 3-deoxy anthocyanins, which have a yellow color due to dihydroxylation of the third carbon, are more stable than red-colored 3-hydroxy anthocyanins. The addition of more hydroxyl groups to the phenyl ring will darken the color, while the replacement of hydroxyl groups with methoxyl groups at the 3′ and 5′ positions enhances the red hue (Amić, Davidović-Amić, & Trinajstić, 1993). Anthocyanins exhibit reduced stability at elevated solution temperatures. A study of Mori, Goto-Yamamoto, Kitayama, and Hashizume (2007) found that heat treatment up to 35 °C significantly decreased total anthocyanin content in common grapes compared to control berries at 25 °C. However, heat treatment of anthocyanin-rich extracts may not lead to pigment degradation, as these extracts typically contain phenolic compounds that inhibit enzymatic degradation by polyphenol oxidase. Moreover, mild heat treatment, such as blanching, in the food processing industry has been demonstrated to prevent anthocyanin oxidation by inactivating the enzymatic reaction up to 50 °C (Patras, Brunton, & O'Donnell, 2010). The primary anthocyanins found in *C. ternatea* are ternatins, which are characterized by their acylation and glycosylation patterns. Acyl groups in these anthocyanins form intramolecular bonds that enhance structural stability and result in deeper, more stable colors. They stabilize the chromophore, making the pigments less susceptible to color loss under varying pH levels and light exposure (Bakowska-Barczak, 2005). Additionally, acyl groups act as barriers against enzymatic degradation and oxidation, which commonly affect non-acylated anthocyanins (Houghton, Appelhagen, & Martin, 2021). This finding provides valuable insights into the stability of natural colorants derived from butterfly pea extract, offering understanding of colorant changes during storage. Such information is crucial for implementing these colorants in the food and beverage industries, especially considering the factors of storage time and conditions.

### 3.9 Impact of BPF extract concentration in coconut milk jelly on the color properties

The different concentration (0, 0.5, 1, 2, and 5% *w*/w) of BPF extract as food colorant in coconut milk jelly products was investigated. In this experiment, the sample achieved a final pH of 6.5. It was observed that as the concentration increased, the coconut milk jelly product became darker, resulting in reduced brightness (lower L* value). Additionally, the jelly product displayed a deeper green hue (lower a* value) with the lowest a* value occurring at a 1% concentration, leading to a bluer color (increased b* value). Table 5 illustrates that the jelly product exhibits a deeper blue color with an increase in BPF extract concentration. Given the importance of color appearance for consumer acceptance in dessert, it becomes crucial to investigate the suitability of adding colorants to food products. Anthocyanin pigments are a valuable natural food colorant but are susceptible to temperature-induced instability. A study of Thuy, Ben, Ngoc, and Tai (2022) found that the Vietnamese traditional rice ball had high sensory value and low constant rate of anthocyanin degradation when adding 10% BPF extract. It also reported that heat and processing lead to anthocyanin loss in the foods. Higher extract utilization ratios are associated with better anthocyanin retention under the same heating time. Steaming processes, as observed in black rice, can cause an 88% reduction in anthocyanin content due to thermal degradation (Surh & Koh, 2014). This loss influences the color change observed in cakes containing butterfly pea flower extract after steaming. Thermal processing in the food industry can degrade anthocyanins, and researchers have proposed various pretreatments to increase extraction yield and enhance color pigment stability (Charurungsipong et al., 2020). Temperature was found to influence anthocyanin stability, with higher temperatures accelerating anthocyanin degradation. Olivas-Aguirre et al. (2016) reported that anthocyanin decomposition rates double for every 10 °C increase in storage temperature. It has been reported that cyanidin 3-glucoside and cyanidin 3-rutinoside decompose at 100 °C in weakly acidic solutions (pH 1–4), both in the presence and absence of oxygen. Anthocyanins may further degrade into coumarin derivatives through decomposition or polymerization and may form complex compounds (Li, Zhang, Wang, Feng, & Han, 2023). Chemical pretreatments involve associating anthocyanins with copigments, such as flavonoids, alkaloids, amino acids, organic acids, metals, and phenolics, to protect them from nucleophilic addition of water, preventing the conversion of the flavylium ion into pseudobase or colorless forms (Bakowska-Barczak, Kucharska, & Oszmianski, 2003). Food product color is directly influenced by the concentration of natural colorants. These research findings have practical applications for food businesses, enabling the use of safe natural colorants in coconut milk jelly for Thai traditional food items. This approach enhances product appeal and allows better control over color degradation, aligning with the loss of anthocyanin content during processing and storage. The results emphasize the importance of carefully applying cooking conditions to preserve the natural color of the dessert product.

**Table 5:** Color properties and appearance of the coconut milk jelly product incorporating colorants derived from BPF, ranging from 0 to 5% (*w*/w) concentration.

Color value⁎	Concentration (%)				
	0	0.5	1	2	5
*L*⁎	44.68 ± 1.32a	37.32 ± 1.10b	27.49 ± 1.60c	21.29 ± 1.56d	17.53 ± 1.81d
*a*⁎	−1.00 ± 1.47a	−4.28 ± 1.54a	−9.61 ± 1.56b	−7.71 ± 1.73b	−4.64 ± 0.95a
*b*⁎	−3.52 ± 0.68a	−4.66 ± 1.27a	−13.66 ± 1.42bc	−11.64 ± 1.64b	−16.62 ± 1.50c
Appearance					

Different letters denote significant differences (*p* < 0.05) between concentrations.

⁎ The value represents the mean of three replications with standard error.

### 3.10 Correlation analysis

**Fig. 7:**
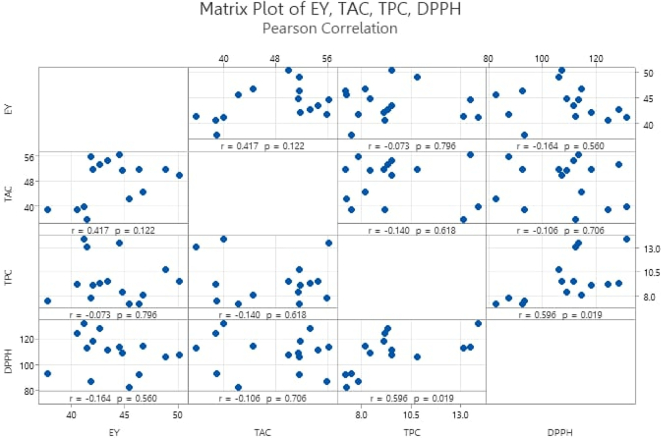
Correlation analysis and matrix plot of EY, TAC, TPC and DPPH from BPF extract. r: correlation coefficient and *p*-value for a 95% confidence interval.

The correlation analysis conducted between various response parameters provides valuable insights into the relationships among these variables, and a matrix plot is shown in Fig. 7. The primary variables analyzed were EY, TAC, TPC, and DPPH. The correlation coefficient between EY and TAC was 0.41661, indicating a moderate positive correlation. Although this correlation was not statistically significant (*p* = 0.1244), it suggests a tendency for higher extraction yields to be associated with higher anthocyanin content. However, the correlation between EY and DPPH antioxidant activity was −0.16379, indicating a weak negative relationship (*p* = 0.5669). This suggests that a higher extraction yield does not necessarily correspond to higher antioxidant activity, potentially due to the presence of non-antioxidant compounds in the extracts or the specific nature of the antioxidant compounds present. A more substantial positive correlation was observed between TPC and DPPH antioxidant activity (0.59644, *p* = 0.0172). This significant correlation indicates that phenolic compounds play a crucial role in the antioxidant activity of the extracts. The strong relationship underscores the importance of phenolics as key contributors to the overall antioxidant capacity of the extracts, validating the use of TPC as an indicator of antioxidant potential in BPF extracts. These findings provide a better understanding of the relationships between the extracted compounds and their bioactivities, informing future optimization and utilization of butterfly pea flower extracts in food applications.

## 4 Conclusions

This study aimed optimal conditions for extracting colorants from BPFs, revealing positive effects of high solid-to-liquid ratio on yield, phenolic compounds, and antioxidants, while ultrasonic extraction time increased anthocyanin content, and ethanol concentration enhanced antioxidant activity. The statistical analysis indicated that the linear and quadratic terms of these variables significantly influenced the increased pigment extraction from BPF, achieved through ultrasound treatment using the BBD method. The optimal conditions, as determined by the quadratic polynomial equation, include a 1:40 (g:mL) of sample-to-solvent ratio, 37 min of extraction time, and 61% ethanol concentration. The model's reliability was confirmed through close agreement between predicted and actual values. BPF extract showed a potential as a natural and appealing food coloring agent through incorporation into a coconut milk jelly product. The optimized conditions could be applied for producing colorants used in the Thai dessert industry.

## CRediT authorship contribution statement

**Samart Sai-Ut:** Writing – original draft, Visualization, Supervision, Conceptualization. **Apisara Teksee:** Validation, Methodology, Formal analysis. **Jaksuma Pongsetkul:** Writing – review & editing. **Sirima Sinthusamran:** Writing – review & editing. **Saroat Rawdkuen:** Writing – review & editing, Supervision.

## Declaration of competing interest

The authors declare that they have no known competing financial interests or personal relationships that could have appeared to influence the work reported in this paper.

## Data Availability

No data was used for the research described in the article.
